# Cold–Temperate *Betula platyphylla* Sukaczev Forest Can Provide More Soil Nutrients to Increase Microbial Alpha Diversity and Microbial Necromass Carbon

**DOI:** 10.3390/microorganisms13102291

**Published:** 2025-10-01

**Authors:** Yunbing Jiang, Mingliang Gao, Libin Yang, Zhichao Cheng, Siyuan Liu, Yongzhi Liu

**Affiliations:** 1 Key Laboratory of Biodiversity, Institute of Natural Resources and Ecology, Heilongjiang Academy of Sciences, Harbin 150040, China; xingantiger1998@163.com (Y.J.); chengzc928@163.com (Z.C.); liuliu9826@163.com (S.L.); 2 College of Geographical Science, Harbin Normal University, Harbin 150025, China; 3 Heilongjiang Huzhong National Nature Reserve, Huzhong 165038, China; zrbhjzhk@163.com

**Keywords:** forest types, PLFAs, microbial necromass carbon, community structure

## Abstract

Changes in vegetation type shape the soil microenvironment, thereby regulating the changes in the organic carbon pool by influencing microbial communities and the accumulation of microbial necromass carbon (MNC). This study investigated microbial biomass—via phospholipid fatty acids (PLFAs) analysis—and MNC accumulation across three cold–temperate forest types: *Larix gmelinii* forest (L), *Larix gmelinii*–*Betula platyphylla* Sukaczev mixed forest (LB), and *Betula platyphylla* Sukaczev forest (B). The results showed that the L had the lowest contents of pH, water content (WC), soil organic carbon (SOC), total nitrogen (TN), available nitrogen (AN), and total phosphorus (TP), but the highest contents of dissolved organic carbon (DOC), available phosphorus (AP), and carbon to nitrogen ratio (C/N) (*p* < 0.05). LB had the lowest PLFAs content and the highest ratio of Gram-positive bacteria/Gram-negative bacteria (G+/G−), and total fungi/total bacteriai (F/B) of L was the highest. B had the highest alpha diversity index, and significantly positively correlated with pH, SOC, TN, AN, and TP. TP and C/N were the primary elements for significant differences in microbial community structure. The order of MNC content and its contribution to SOC was B > LB > L. MNC was significantly negatively correlated with PLFAs, DOC, and AP, and significantly positively correlated with pH, SOC, TN, AN, TP, Shannon–Wiener and Pielou indices. In conclusion, this study demonstrates that *Betula platyphylla* Sukaczev forest retains more carbon, nitrogen, and phosphorus, microbial alpha diversity, and acquires more MNC, which can provide a basis for subsequent forest management and carbon sequestration projects.

## 1. Introduction

Forest soil stores 16–26% of the global soil carbon pools, playing a critical role in regulating global carbon cycles and mitigating climate change [1]. Microbial necromass carbon (MNC), a stable component of soil organic carbon (SOC), contributes 30 to 60% of SOC and is considered a major contributor to the formation of long-term stable carbon pools [2,3]. The microbial carbon pump theory proposes that plant-derived components are the primary source of microbial necromass carbon accumulation, which is subsequently transformed into MNC through both microbial internal turnover and external modification processes [3]. Against the backdrop of global climate warming and growing human demand, global forest fragmentation has intensified worldwide. Threatened tree species are experiencing survival crises [4], natural forest areas have drastically shrunk [5], and the southern boundary of coniferous forests has shifted northward, with broadleaf forests gradually expanding into coniferous forest areas [6].

Microorganisms, representing the most biologically active component of soil, play a critical role not only in decomposing forest litter and mediating greenhouse gas emissions, but also in the formation and stabilization of soil organic carbon [3]. Changes in forest type can alter the input of plant litter and root exudates, thereby influencing the stability of SOC pools by affecting microorganisms [7]. Comparative studies indicated that MNC content was higher in northern and temperate forest soils than in tropical and subtropical regions [1]. Li et al. [7] reported that MNC content in artificial forest soils is significantly lower than in protected forests, with a strong positive correlation observed between MNC and phospholipid fatty acids (PLFAs) content. Furthermore, high-quality litter from broadleaf forests is more quickly decomposed by soil microorganisms into humus, leading to greater SOC accumulation compared to coniferous litter [8]. Although early study shows that soil amino sugar content is significantly higher in broadleaf and mixed forests than in coniferous forests [9], another study found no significant differences among the three types [10]. Meanwhile, changes in forest type also affect the community composition and diversity of soil microorganisms [11]. In general, coniferous forest litter contains more recalcitrant compounds such as lignin and tannins, resulting in a slower decomposition rate [12]. In contrast, broadleaf forest litter supports faster nutrient release, promoting greater microbial diversity and activity [13]. Therefore, broadleaf forests exhibit higher microbial diversity and richness, and there are significant differences in dominant microorganisms between the two forest types [14]. Additionally, mixed forests often exhibit faster litter decomposition rates than pure forests and rapid nutrient input offers more substrate for increased microbial biomass and diversity [15]. Nevertheless, some studies reported higher microbial alpha diversity in pure forests than in mixed forests [16], or identified no significant difference [17]. Thus, there remains controversy regarding the changes in soil microbial communities and MNC content between pure and mixed forests.

The Greater Khingan Mountains harbor China’s only cold–temperate coniferous forest, an ecosystem highly vulnerable to climate change. *Larix gmelinii* serves as the predominant constructive species in these forests and represents the climax community under local cold–temperate conditions. *Betula platyphylla* Sukaczev forests (B) are the dominant species during the pioneer stage of secondary succession and gradually transition into *Larix gmelinii*–*Betula platyphylla* Sukaczev mixed forests (LB) and *Larix gmelinii* forests (L) over time. Under the background of climate warming, the southern boundary of L is shifting northward, and the B is gradually expanding northward [18], leading to significant changes in forest types. Currently, previous studies have examined soil microbial communities in different forest types within the cold–temperate forest types of the Greater Khingan Mountains, primarily focusing on the L [19] and B [20]. But there have been few comparative investigations of mixed forests with pure forests, limiting our understanding of the adaptation processes of soil microbial communities in the cold–temperate forests in response to vegetation changes. Moreover, changes in forest types represent variations in carbon input, which influence not only microbial biomass and community structure but also the accumulation of microbial necromass carbon [7]. However, the content of MNC and its contribution to SOC accumulation across different forest types in this region remain unexplored. Therefore, this study aims to (1) compare the differences in soil microbial community composition and biomass across different forest types; and (2) quantify the contribution of MNC to SOC and identify its driving factors, including the relationship with microbial PLFAs. We hypothesize that (1) differences in soil nutrient content across forest types will result in significant variations in microbial biomass and distinct community compositions; and (2) both the content of MNC and its contribution to SOC may increase with higher soil nutrient levels and concentrations of living microorganisms.

## 2. Materials and Methods

### 2.1. Study Site

**Figure 1:**
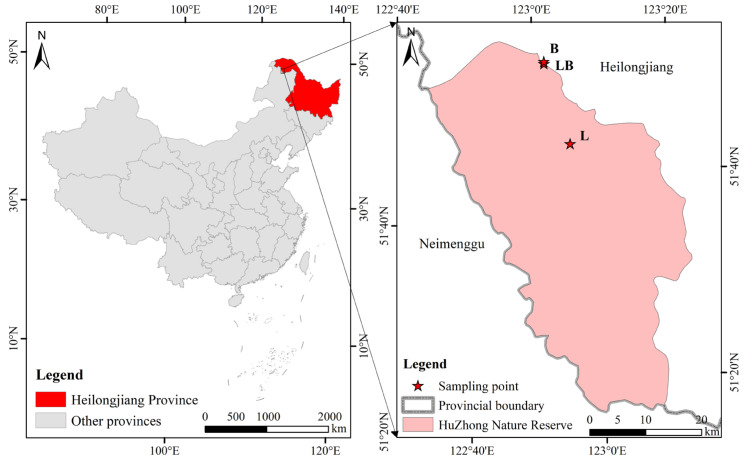
Geographical location of sampling sites in this study. Note: L: *Larix gmelinii* forest; LB: *Larix gmelinii*–*Betula platyphylla* Sukaczev mixed forest; B: *Betula platyphylla* Sukaczev forest.

The study site is located in the Huzhong National Nature Reserve in the Greater Khingan Mountains, China (51°49′01′′ N–51°49′19″ N, 122°59′33″ E–123°00′03″ E) (Figure 1). This area belongs to the cold–temperate continental monsoon climate, with an average annual temperature and precipitation of −4 °C and 458.3 mm, respectively. In addition, the region also belongs to the mid–high latitude permafrost region, and permafrost is widely developed. The *Larix gmelinii* forest is a typical dominant plant community, with *Betula platyphylla* Sukaczev forest being the primary companion species in *Larix gmelinii* forests. *Rhododendro dauricum* and *Ledum palustre* are the most common shrubs, and *Maianthemum bifolium* and *Carex appendiculata* are the most common herbs [21].

### 2.2. Soil Sample Collection and Treatment

In August 2024, three forest types that have not been cut down or burned were selected in the China National Nature Reserve: *Larix gmelinii* forest (L), *Larix gmelinii*–*Betula platyphylla* Sukaczev mixed forest (LB), and *Betula platyphylla* Sukaczev forest (B). Each forest type was established in three 20 m × 20 m plots, with five 1 m × 1 m small plots chosen using the 5-point sample approach. Soil samples were collected from the 0−20 cm layer after removing humus. All soil samples from the same forest type were mixed uniformly after removing stones and roots on site, and the soil weight in each forest type exceeded 3 kg. Finally, the samples were transported back to the laboratory. In the laboratory, the soil was divided into two parts: one part was stored at −20 °C for microbial community analysis, and the other part was used for soil physicochemical properties determination.

### 2.3. Analyses of Soil Physicochemical Properties

Moisture content (WC) was determined using the drying method. Soil pH was measured using a combination electrode (PB–10, Sartorius, Gottingen, Germany) under a 1:10 (*m*/*v*) condition. Soil total nitrogen (TN) content was determined using an elemental analyzer (Elementar Vario El III, Hanau, Germany). Available nitrogen (AN) content was measured using a flow injection analyzer (San++, Skalar, Delft, The Netherlands). The content of soil organic carbon (SOC) and dissolved organic carbon (DOC) was measured using a total organic carbon analyzer (Vario TOC cube, Elementar, Hanau, Germany) [21]. Total phosphorus (TP) and available phosphorus (AP) content were determined using concentrated sulfuric acid digestion–molybdenum antimony colorimetric method and extraction–molybdenum antimony colorimetric method, respectively [22].

### 2.4. Measurement of Phospholipid Fatty Acids

The method described by Bossio and Scow [23] was used to determine soil phospholipid fatty acids. Weigh 4 g of freeze-dried soil sample and add 22.8 mL of a single-phase mixture of chloroform/methanol/citric acid buffer (1:2:0.8 *v*/*v*/*v*, 0.15 mol L^−1^, pH 4.0) to extract the soil twice, followed by separation of phospholipids, neutral lipids, and glycolipids on a silica column (Supelco, Bellefonte, PA, USA). Before quantifying phospholipid concentrations using a standard curve, add nonadecanoic acid methyl ester (19:0) as an internal standard. After phospholipid methylation, different molecules were separated using a gas chromatography system (GC; N6890, Agilent, Santa Clara, CA, USA), and the components of PLFAs were identified and quantified using MIDI Sherlock microbial identification software (Version 4.5, MIDI, Newark, DE, USA). Phospholipid fatty acid markers for different microbial groups were referenced from Zhang et al. [24]. The diversity formula is as follows:

(1) $$\mathrm{Diversity index of Shannon–Wiener:}\;H=-\sum_{i=0}^{s}P_{i}lnP_{i}$$

(2) $$\mathrm{Dominance index of Simpson:}\;D=1-\sum_{i=1}^{s}{P_{i}}^{2}$$

(3) Evenness index of Plielou: J=H/lnS

where S represents the number of PLFAs biomarkers in the sample, and P_i_ represents the proportion of individuals of the i–characteristic fatty acids to the total characteristic fatty acids.

### 2.5. Measurement of Microbial Necromass Carbon

The method described by Ma et al. [25] was used to determine the amino sugars in the soil to characterize the microbial necromass carbon. In brief, 0.3 mg of freeze-dried sample was added to 10 mL of 6 M HCl and hydrolyzed for 8 h, followed by the addition of 100 µg of inositol, filtration, and drying. Subsequently, the sample was redissolved, centrifuged, and the supernatant was dried with N_2_. Afterwards, add 1 mL of deionized water and 100 µg of N-methylglucosamine, mix thoroughly, and dry. Then, add 0.3 mL of derivatization reagent (a 4:1 solution of pyridine and methanol, hydroxylamine hydrochloride, and 4-dimethylaminopyridine), cool, and add 1 mL of acetic anhydride. Finally, extract the derivatives from the aqueous solution with dichloromethane, dry under N_2_, dissolve in 200 μL of ethyl acetate-hexane mixed solvent, and transfer to a gas chromatography (GC 7890A, Agilent Technologies Inc., Santa Clara, CA, USA) determination vial to determine the content of glucosamine (GluN) and muramic acid (MurA). The calculation of fungal necromass carbon (FNC), bacterial necromass carbon (BNC), and MNC followed the method described by Wang et al. [2].

(4) $$FNC=(\frac{GluN}{179.17}-2\times \frac{MurA}{251.23})\times 179.17\times 9$$

(5) BNC=MurA×45

(6) MNC=FNC+BNC

where 179.17 and 251.23 are the molecular weights of GluN and MurA, respectively; 9 is the conversion value of fungal-derived glucosamine to fungal necromass carbon, and 11 is the conversion value of MurA to bacterial necromass carbon. FNC: fungal necromass carbon; BNC: bacterial necromass carbon; MNC: microbial necromass carbon.

### 2.6. Statistical Analyses

Based on IBM SPSS Statistics 25.0 (SPSS Inc., Chicago, IL, USA), all experimental data were assessed for normality and homogeneity of variance. For data exhibiting heterogeneous variance, the Games–Howell test was applied, while one-way analysis of variance (ANOVA) was used for data with homogeneous variance. Tukey’s test was used to analyze the differences in soil physicochemical properties, microbial biomass, alpha diversity index, and microbial necromass carbon, and results were presented as mean ± standard deviation (SD). Bar charts and linear fitting graphs were plotted using Origin 2024 (Origin Software Inc., Northampton, MA, USA). Based on R 4.0.2, principal component analysis (PCA) was used in combination with the permutational MANOVA (Adonis) test to analyze the differences in microbial community structure, and a heatmap was used to explore the correlation between microbial community PLFAs, alpha diversity index, and microbial necromass carbon content with soil physicochemical properties based on Spearman correlation. Finally, redundancy analysis (RDA) was completed based on Canoco 5 (Biometris, Groesbeek, Gelderland, The Netherlands).

## 3. Results

### 3.1. Soil Physicochemical Properties in Different Forest Types

**Figure 2:**
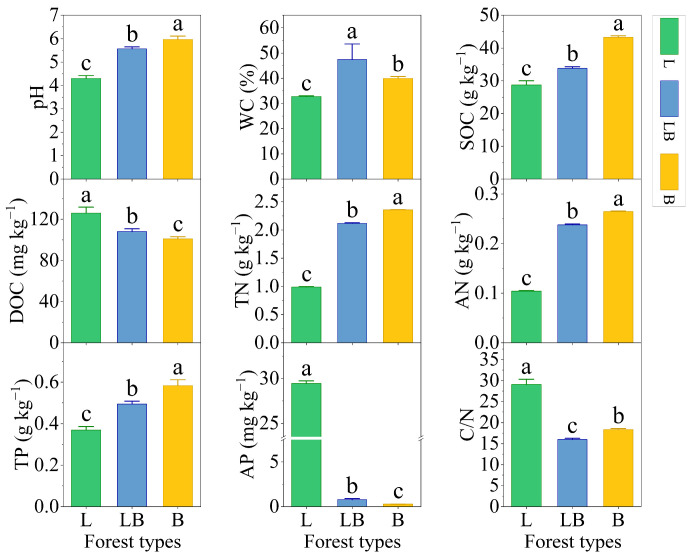
Soil physicochemical properties in different forest types. Note: Data are represented as mean ± SD (*n* = 5), error bars represent the standard deviation; L: *Larix gmelinii* forest; LB: *Larix gmelinii*–*Betula platyphylla* Sukaczev mixed forest; B: *Betula platyphylla* Sukaczev forest. WC: moisture content; TN: total nitrogen; AN: available nitrogen; SOC: soil organic carbon; DOC: dissolved organic carbon; TP: total phosphorus; AP: available phosphorus; C/N: SOC/TN. Different lowercase letters in the same soil physicochemical factor indicate significant differences (*p* < 0.05).

Significant differences were observed in the physicochemical properties of soils across different forest types (Figure 2, *p* < 0.05). The content rankings for pH, SOC, TN, AN, and TP were B > LB > L, while those for DOC and AP were L > LB > B (*p* < 0.05). The content ranking for WC was LB > B > L, and that for C/N was L > B > LB (*p* < 0.05).

### 3.2. Soil Microbial Biomass in Different Forest Types

**Figure 3:**
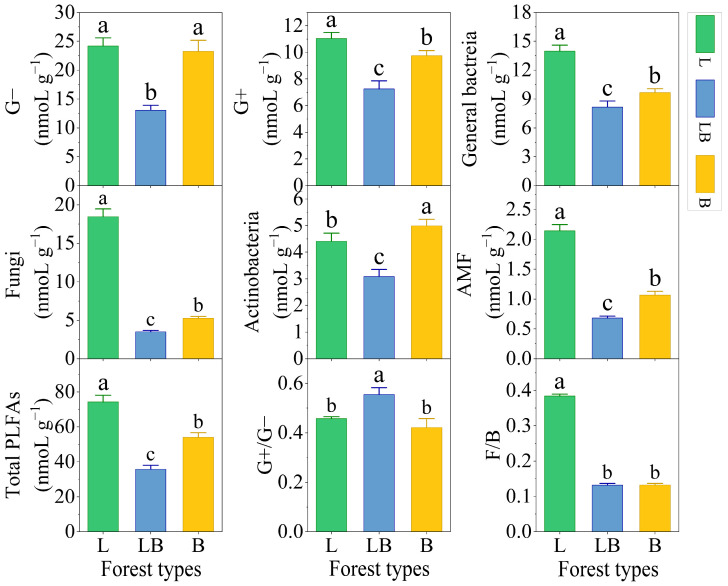
Content of PLFAs in different forest types. Note: Data are represented as mean ± SD (*n* = 5), error bars represent the standard deviation; G−: Gram-negative bacteria; G+: Gram-positive bacteria; AMF: arbuscular mycorrhizal fungi; total PLFAs: sum of the PLFAs content of G−, G+, general bacteria, fungi, Actinobacteria, and AMF; F/B: (sum of the PLFAs content of G−, G+, general bacteria and Actinobacteria)/(fungi + AMF). L: *Larix gmelinii* forest; LB: *Larix gmelinii*–*Betula platyphylla* Sukaczev mixed forest; B: *Betula platyphylla* Sukaczev forest. Different lowercase letters in the same phospholipid fatty acid markers or ratios indicate significant differences (*p* < 0.05).

There were significant differences in the content of soil Gram-positive bacteria (G+), general bacteria, fungi, arbuscular mycorrhizal fungi (AMF), and total PLFAs among different forest types, with the content ranking being L > B > LB (*p* < 0.05), while the content ranking of Actinomycetes was B > L > LB (Figure 3, *p* < 0.05). Gram-negative bacteria (G−) content was significantly lower in the LB than in the L and B. The ratio of Gram-positive bacteria/Gram-negative bacteria (G+/G−) was significantly higher in the LB than in the L and B, and total fungi/total bacteria (F/B) was significantly higher in the L than in the LB and B (*p* < 0.05).

### 3.3. Soil Microbial Community Diversity and Structure in Different Forest Types

**Figure 4:**
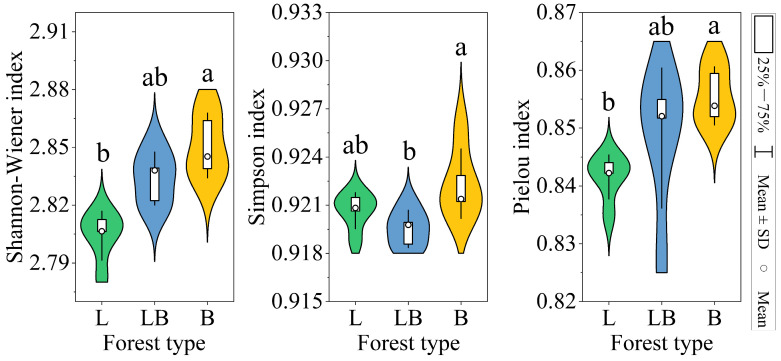
Soil microbial diversity indices in different forest types. Note: L: data are represented as mean ± SD (*n* = 5), error bars represent the standard deviation; *Larix gmelinii* forest; LB: *Larix gmelinii*–*Betula platyphylla* Sukaczev mixed forest; B: *Betula platyphylla* Sukaczev forest. Different lowercase letters in the same index indicate significant differences (*p* < 0.05).

**Figure 5:**
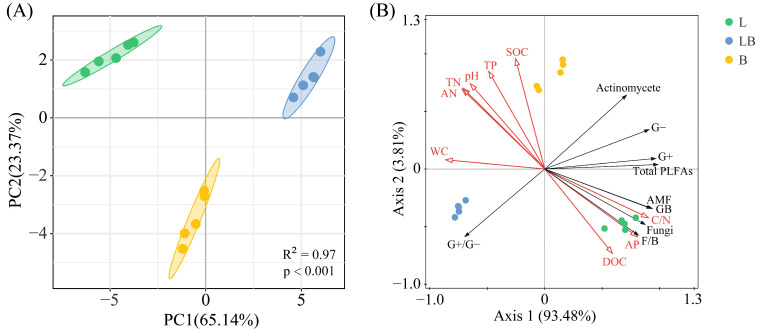
PCA (**A**) and RDA (**B**) analyses of soil microbial communities in different forest types. Note: The shaded ellipse indicates the range of the 95% confidence interval. L: *Larix gmelinii* forest; LB: *Larix gmelinii*–*Betula platyphylla* Sukaczev mixed forest; B: *Betula platyphylla* Sukaczev forest. WC: moisture content; TN: total nitrogen; AN: available nitrogen; SOC: soil organic carbon; DOC: dissolved organic carbon; TP: total phosphorus; AP: available phosphorus; C/N: SOC/TN. G−: Gram-negative bacteria; G+: Gram-positive bacteria; AMF: arbuscular mycorrhizal fungi; total PLFAs: sum of the PLFAs content of G−, G+, general bacteria, fungi, Actinobacteria, and AMF; F/B: (sum of the PLFAs content of G−, G+, general bacteria and Actinobacteria)/(fungi + AMF).

The Shannon–Wiener index and Pielou index in B were significantly higher than those of the L, while the Simpson index in B was significantly higher than in the LB (Figure 4, *p* < 0.05). The results of the PCA and Adonis test showed that the composition of the soil microbial community varied significantly among the various forest types (Figure 5A, *p* < 0.001). The two principal components related to microbial communities cumulatively contributed 88.51%, with PC1 and PC2 explaining 65.14% and 23.37%, respectively (Figure 5B). RDA results revealed that nine physicochemical factors collectively explained 97.29% of the variation in microbial community composition, with Axis 1 and Axis 2 explaining 93.48% and 3.81% of the variation, respectively (Figure 5B). C/N (76.6%, *p* < 0.01) and TP (17.8%, *p* < 0.01) were the primary factors influencing microbial community composition (Table 1).

### 3.4. Soil Microbial Necromass Carbon in Different Forest Types

**Figure 6:**
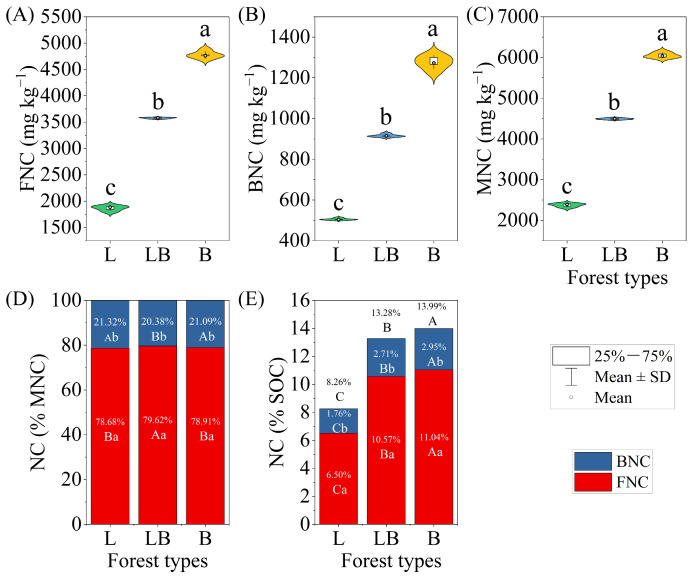
Characteristics of soil microbial necromass carbon content and contribution to SOC in different forest types. Note: Data are represented as mean ± SD (*n* = 5), error bars represent the standard deviation; L: *Larix gmelinii* forest; LB: *Larix gmelinii*–*Betula platyphylla* Sukaczev mixed forest; B: *Betula platyphylla* Sukaczev forest. FNC: fungal necromass carbon; BNC: bacterial necromass carbon; MNC: microbial necromass carbon. Different lowercase letters in (**A**–**C**) indicate significant differences in the content of various indicators between different forest types (*p* < 0.05); different lowercase letters in (**D**,**E**) indicate significant differences in the proportion and content of the same forest type, while different uppercase letters indicate significant differences in the proportion of the same necromass carbon component between different forest types (*p* < 0.05).

Significant differences were found in FNC, BNC, and MNC across the various forest types, with the content rankings for B > LB > L (Figure 6A–C, *p* < 0.05). Within the same forest type, FNC accounted for a significantly higher proportion of MNC and SOC than BNC (Figure 6D,E). Across different forest types, the LB group’s FNC accounted for a significantly higher proportion of MNC than in the L and B, while BNC accounted for a significantly lower proportion of MNC than in the L and B (*p* < 0.05). Additionally, the proportions of FNC, BNC, and MNC in soil SOC differed significantly among forest types, with the order being B > LB > L (*p* < 0.05).

### 3.5. Correlation and Regression Analysis

**Figure 7:**
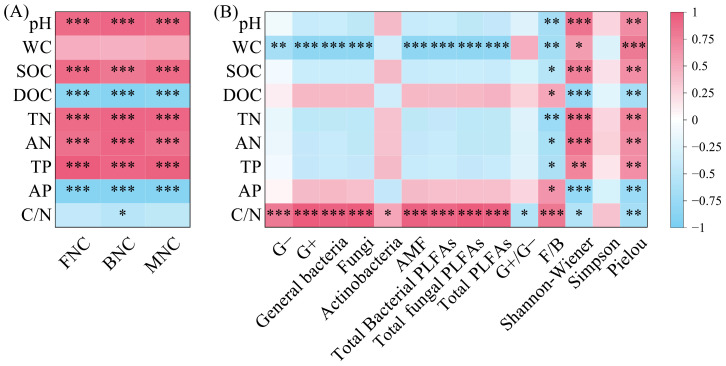
Correlation between microbial necromass carbon (**A**) and PLFAs content of various microbial groups (**B**) and soil physicochemical properties. Note: L: *Larix gmelinii* forest; LB: *Larix gmelinii*–*Betula platyphylla* Sukaczev mixed forest; B: *Betula platyphylla* Sukaczev forest. FNC: fungal necromass carbon; BNC: bacterial necromass carbon; MNC: microbial necromass carbon. WC: moisture content; TN: total nitrogen; AN: available nitrogen; SOC: soil organic carbon; DOC: dissolved organic carbon; TP: total phosphorus; AP: available phosphorus. G−: Gram-negative bacteria; G+: Gram-positive bacteria; AMF: arbuscular mycorrhizal fungi; total PLFAs: sum of the PLFAs content of G−, G+, general bacteria, fungi, Actinobacteria, and AMF; F/B: (sum of the PLFAs content of G−, G+, general bacteria and Actinobacteria)/(fungi + AMF). * *p* < 0.05; ** *p* < 0.01; *** *p* < 0.001.

BNC, FNC, and MNC were all significantly positively correlated with pH, SOC, TN, AN, and TP, and significantly negatively correlated with DOC and AP (*p* < 0.001); BNC was significantly negatively correlated with C/N (Figure 7A, *p* < 0.05). PLFAs content in all taxonomic groups was significantly positively correlated with C/N (Figure 7B, *p* < 0.05). Except for Actinobacteria, PLFAs content in other taxonomic groups was significantly negatively correlated with WC (*p* < 0.01). G+/G− was significantly negatively correlated with C/N, while F/B was significantly negatively correlated with pH, SOC, TN, AN, and TP, and significantly positively correlated with DOC, AP, and C/N (*p* < 0.01). The Shannon–Wiener index and Pielou index were significantly positively correlated with pH, WC, SOC, TN, AN, and TP, and significantly negatively correlated with DOC, AP, and C/N (*p* < 0.05).

**Figure 8:**
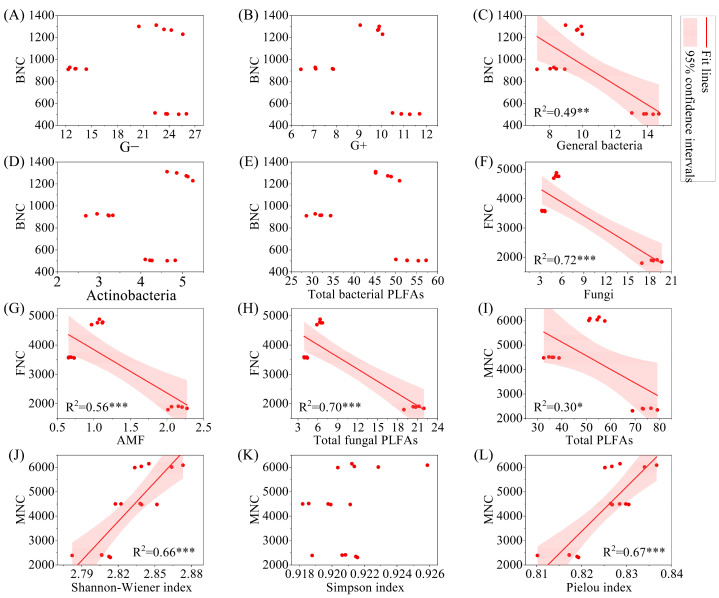
Regression analysis of microbial necromass carbon and PLFAs content and microbial alpha diversity index. Note: (**A**–**E**): relationship between BNC and G−, G+, general bacteria, Actinobacteria, and total bacterial PLFAs; (**F**–**H**): relationship between FNC and fungi, AMF, and total fungal PLFAs; (**I**–**L**): relationship between MNC and total PLFAs, shannon-wiener index, simpson index, and pielou index. G−: Gram-negative bacteria; G+: Gram-positive bacteria; AMF: arbuscular mycorrhizal fungi; total PLFAs: sum of the PLFAs content of G−, G+, general bacteria, fungi, Actinobacteria, and AMF; F/B: (sum of the PLFAs content of G−, G+, general bacteria and Actinobacteria)/(fungi + AMF). FNC: fungal necromass carbon; BNC: bacterial necromass carbon; MNC: microbial necromass carbon. * *p* < 0.05; ** *p* < 0.01; *** *p* < 0.001.

The results of linear regression analysis indicated that BNC was significantly negatively correlated with general bacteria (Figure 8, *p* < 0.01), while FNC was significantly negatively correlated with fungi, AMF, and total fungal PLFAs (*p* < 0.001). MNC was significantly negatively correlated with total PLFAs (*p* < 0.05). From the perspective of significant correlations, BNC, FNC, and MNC all showed a significant decrease with increasing biomass (*p* < 0.05).

## 4. Discussion

### 4.1. Changes in Soil Physicochemical Properties in Different Forest Types

Variations in litter sources, decomposition rates, and root exudates among different forest types significantly influence soil physicochemical properties [9]. This study found that the highest values of pH, SOC, TN, AN, and TP were observed in the B, followed by the LB and L, while the C/N ratio was higher in the L than in the other two forest types. The primary reason is that coniferous litter contains fewer nutrients and is rich in lignin and phenolic compounds, whereas broadleaf litter decomposes more rapidly, contributing relatively higher inputs of soil nutrients such as SOC, thereby elevating nutrient availability and reducing soil C/N ratios [26]. Second, the decomposition process of coniferous litter and the absorption of nutrients by coniferous tree roots will release more organic acid compounds into the soil [27,28]. This process accelerates the consumption of salt-based ion pools (Ca^2+^, Mg^2+^, and K^+^) and exacerbates soil acidification [29]. Conversely, broadleaf litter decomposition produces more base ions, which neutralize soil H^+^ [30], resulting in the lowest pH in the L and the highest in the B. The soil moisture content in the LB was significantly higher than in the L and B, consistent with earlier studies showing that mixed forests have significantly higher moisture content than pure forests [31]. This is mainly attributed to the fact that mixed forests improve soil structure, increase soil porosity, and thereby enhance soil water storage capacity [32]. DOC is a readily metabolizable carbon source for microorganisms, mainly derived from the leaching of litter and plant residues, root exudates, and the decomposition of SOC. However, there is no consensus on the levels of DOC content in coniferous and broadleaf forests. For example, Deng showed that broadleaf forest soils contain more DOC than coniferous forest soils [33], but Tian and Man [34] and our study discovered that coniferous forest DOC content was higher than that of broadleaf forest. The explanation for the difference is the more aromatic character of DOC generated by coniferous litter and root systems, which is more difficult to decompose than DOC in broadleaf forest soil, resulting in more accumulation of DOC in coniferous forests [13]. Additionally, AP in the L soils was significantly higher than in the LB and B, primarily because the L soils have the lowest pH, and acidic conditions, accelerating the dissolution of iron-aluminum phosphates, enhancing AP release [12]. In summary, this study demonstrates that the *Betula platyphylla* Sukaczev forest accumulates more soil carbon, nitrogen, and phosphorus than the *Larix gmelinii* forest and the *Larix gmelinii*–*Betula platyphylla* Sukaczev mixed forest, which can provide foundational data for future forest management strategies.

### 4.2. Factors Influencing Changes in the Soil Microbial Community

In this study, the G+, general bacteria, fungi, AMF, and total PLFAs contents in the L were significantly higher than those in the LB and B, while the PLFAs contents of all taxa in the LB were significantly lower than those in the L and B. The main reasons for this are as follows. (1) The high available nutrient content of soil stimulates microbial growth. The L soils exhibited the highest DOC and AP concentrations, providing the most available carbon sources for soil microorganisms [35], and phosphorus elements essential for microbial production of nucleic acids and phospholipids [36]. (2) Competition within mixed forest plants may cause available nutrients in the soil to be prioritized for plant consumption [37], thereby limiting nutrient accessibility for microorganisms and ultimately reducing microbial biomass. (3) High moisture content inhibits soil respiration and metabolic activity, and we found the lowest moisture content in L soils and the highest in LB. Heatmap analysis revealed that the PLFAs content of all taxa was strongly negatively correlated with WC, suggesting that higher moisture content may inhibit microbial survival by reducing soil oxygen supply [38]. Furthermore, given the critical role of fungi in decomposing recalcitrant compounds such as lignin [39], the content of fungi in the L forests with the highest C/N content (the most difficult to decompose substances) is higher than that in other forest types. Meanwhile, this is also the key explanation for the highest F/B ratio in the L soils. The LB soils have the lowest PLFAs concentration for G+ and G−, but the highest G+/G− ratio compared to other forest types. This shift may be attributed to intense plant competition within mixed forests, where root systems preferentially consume labile carbon sources, causing a relative reduction in easily available carbon, ultimately leading to G+ being more suitable for this resource-limited condition [37].

This study found that the L’s Shannon–Wiener and Pielou indices were significantly lower than in the LB and B. This was mainly attributed to the fact that the nutrient composition of the L soil is difficult to decompose and utilize by most microorganisms. Only a small number of microorganisms can dominate under conditions of limited nutrient availability, leading to high dominance and reduced microbial diversity and evenness [40]. In addition, the B soils exhibited higher SOC, TN, and TP storage, which provide abundant substrates for microbial growth and reproduction, thereby contributing to the maintenance of high soil microbial alpha diversity [41]. Combining PCA and RDA results revealed significant differences in soil microbial community composition among different forest types, with C/N and TP identified as the main influencing factors. These results suggest that divergent soil environments, particularly soil nutrient conditions, shape distinct microbial community structures [42]. C/N is widely recognized as a key indicator of soil organic matter quality and exerts considerable influence on microbial reproduction and metabolism [43]. Generally, soil with higher C/N is more suitable for the growth of fungal communities, while lower C/N favors bacterial community colonization and growth. These results are consistent with the present study, which observed that soil F/B of the L with the highest C/N was significantly higher than in other forest types, demonstrating that C/N is an essential factor influencing the composition of microbial communities [44]. Additionally, phosphorus was identified as a limiting factor for soil microorganisms in tropical forests [45] and the permafrost regions of the Qinghai–Tibet Plateau [46]. Emerging evidence indicates that phosphorus deficiency exists in forest soils of cold–temperate permafrost regions [47], indirectly suggesting that the growth and reproduction of microorganisms in this region are limited by phosphorus, thereby affecting microbial community composition. In summary, the research results support our first hypothesis that changes in forest type can modify the soil environment, thereby influencing soil microbial biomass, community structure, and diversity.

### 4.3. Differences in Content of Soil Microbial Necromass Carbon and Influencing Factors

Low temperatures and freezing conditions restrict the production of microbial necromass carbon in the high-latitude permafrost regions [48]. In this study, both MNC content and MNC/SOC in the 0–20 cm soil layer across the three forest types were significantly lower than the average values reported in earlier studies (>10 g·kg^−1^; 35%) [2,3]. This discrepancy can be attributed to the location of the study area within the cold–temperate permafrost regions of China, where perennial low temperatures and a short growing season constrain both primary and secondary productivity [49] and microbial activity [50]. These limitations reduce the initial energy and driving force of the microbial carbon pump, ultimately decreasing MNC content and its contribution to SOC accumulation. Among the forest types, B exhibited the highest MNC content, followed by LB, while L had the lowest. This pattern reflects the limited microbial utilization of recalcitrant compounds—such as lignin and tannins—derived from *Larix gmelinii* forest, resulting in reduced MNC formation [13]. Across ecosystems, FNC typically accounts for over 65% of MNC and contributes more significantly to SOC than BNC [2], highlighting the central role of fungi in stable carbon sequestration. This study is consistent with those findings, likely due to the fungi being more capable than bacteria at decomposing forest humus and acquiring nutrients [51], as well as the fact that FNC is more stable than BNC [52].

The accumulation of MNC is regulated by a range of biotic and abiotic factors [53]. Angst et al. [54] discovered that microbial alpha diversity is closely related to MNC and a significant predictor of MNC accumulation, which is consistent with the findings of this study. Some investigations have shown that PLFAs content serves as an instantaneous indicator for predicting MNC, and exhibits a significant positive correlation with MNC [55]. However, this study found that soil MNC is not significantly correlated or is significantly negatively correlated with PLFAs, suggesting that instantaneous PLFAs content cannot serve as a reliable predictor of MNC in cold–temperate forest ecosystems. One probable explanation is that coniferous forests have poorer carbon utilization efficiency than broadleaf forests [56], leading to reduced conversion of carbon into microbial biomass and consequently lower MNC production. Secondly, compared to the B, nutrients in litter and root exudates in the L were more difficult for microorganisms to decompose and utilize, due to the MNC itself containing a large amount of carbon and nitrogen nutrients, which can be reused by microorganisms as an effective energy source [57], thereby hindering the accumulation of MNC in L soils. Moreover, FNC, BNC, and MNC were significantly positively correlated with factors such as soil pH and SOC, but significantly negatively correlated with DOC and AP. Some reports indicate that low pH suppresses microbial activity and thereby promotes MNC accumulation by slowing decomposition [58]. But this is not consistent with the results of this study; a more plausible explanation is that under acidic conditions, microbes may allocate more carbon to maintenance respiration rather than growth, reducing necromass production [59]. DOC and AP, as widely accessible nutrients, do not directly act on microbial necromass carbon but rather indirectly influence its concentration by altering microbial growth and metabolism [60]. Additionally, the accumulation of soil MNC is a long-term dynamic process, and DOC and AP exhibit a lag impact on MNC generation [61], or MNC production is insufficient to offset decomposition, leading to a negative correlation. The above findings partially support our second hypothesis, which states that increased soil nutrient content encourages the accumulation of MNC, but there is no substantial positive link between microbial biomass and MNC.

## 5. Conclusions

This study indicated that of the three forest types in the cold–temperate zone, *Betula platyphylla* Sukaczev forest soil conserved more carbon, nitrogen, and phosphorus nutrients and had higher microbial necromass carbon content and microbial alpha diversity, while *Larix gmelinii* forest had the highest total PLFAs content. C/N and TP are the primary factors influencing differences in microbial community composition, while WC and C/N are co-factors influencing microbial PLFAs content. Microbial necromass carbon content is largely influenced by both soil physicochemical properties, microbial biomass, and alpha diversity. The findings of this study provide important data references for forest management and carbon sink research in cold–temperate regions in China.

## Figures and Tables

**Table 1:** Significance test results of RDA.

Factors	Explains (%)	*p*
C/N	76.6	<0.01
TP	17.8	<0.01
pH	1.0	>0.05
AP	0.6	>0.05
SOC	0.8	>0.05
AN	0.4	>0.05
DOC	<0.1	>0.05
TN	<0.1	>0.05
WC	<0.1	>0.05

Note: WC: moisture content; TN: total nitrogen; AN: available nitrogen; SOC: soil organic carbon; DOC: dissolved organic carbon; TP: total phosphorus; AP: available phosphorus; C/N: SOC/TN.

## Data Availability

The original contributions presented in this study are included in the article. Further inquiries can be directed to the corresponding authors.

## References

[1] Xu S., Song X., Zeng H., Wang J. (2024). Soil microbial necromass carbon in forests: A global synthesis of patterns and controlling factors. Soil Ecol. Lett..

[2] Wang B., An S., Liang C., Liu Y., Kuzyakov Y. (2021). Microbial necromass as the source of soil organic carbon in global ecosystems. Soil Biol. Biochem..

[3] Liang C., Schimel J.P., Jastrow J.D. (2017). The importance of anabolism in microbial control over soil carbon storage. Nat. Microbiol..

[4] Cabrera-Ariza A.M., Rivas C.A., Aguilera-Peralta M., Navarro-Cerrillo R.M., Santelices-Moya R. (2025). Updating the distribution of *Nothofagus alessandrii*: Impact of deforestation, fragmentation and connectivity. For. Ecosyst..

[5] Amaral S., Metzger J.P., Rosa M., Adorno B.V., Gonçalves G.C., Pinto L.F.G. (2025). Alarming patterns of mature forest loss in the Brazilian Atlantic Forest. Nat. Sustain..

[6] Mekonnen Z.A., Riley W.J., Randerson J.T., Grant R.F., Rogers B.M. (2019). Expansion of high-latitude deciduous forests driven by interactions between climate warming and fire. Nat. Plants.

[7] Cotrufo M.F., Wallenstein M.D., Boot C.M., Denef K., Paul E. (2013). The Microbial Efficiency-Matrix Stabilization (MEMS) framework integrates plant litter decomposition with soil organic matter stabilization: Do labile plant inputs form stable soil organic matter?. Glob. Change Biol..

[8] Hatton P., Castanha C., Torn M.S., Bird J.A. (2015). Litter type control on soil C and N stabilization dynamics in a temperate forest. Glob. Change Biol..

[9] Dai G., Zhu S., Cai Y., Zhu E., Jia Y., Ji C., Tang Z., Fang J., Feng X. (2022). Plant-derived lipids play a crucial role in forest soil carbon accumulation. Soil Biol. Biochem..

[10] Zhu Y., Hui D., Wang Y.P., Liu F., Huang S., Li J., Zhang L., Chen G., Chen J., Hu Y. (2022). Linking plant lignin components or microbial necromass to soil organic carbon accumulation across different forest types. Res. Sq..

[11] Rodríguez-Rodríguez J.C., Fenton N.J., Bergeron Y., Kembel S.W. (2023). Soil and tree phyllosphere microbial communities differ between coniferous and broadleaf deciduous boreal forests. Plant Soil.

[12] Klotzbücher T., Kaiser K., Stepper C., van Loon E., Gerstberger P., Kalbitz K. (2012). Long-term litter input manipulation effects on production and properties of dissolved organic matter in the forest floor of a Norway spruce stand. Plant Soil.

[13] Chodak M., Beata K., Niklińska M. (2016). Composition and activity of soil microbial communities in different types of temperate forests. Biol. Fert. Soils.

[14] Labouyrie M., Ballabio C., Romero F., Panagos P., Jones A., Schmid M.W., Mikryukov V., Dulya O., Tedersoo L., Bahram M. (2023). Patterns in soil microbial diversity across Europe. Nat. Commun..

[15] Li W., Huang Y., Chen F., Liu Y., Lin X., Zong Y., Wu G., Yu Z., Fang X. (2021). Mixing with broad-leaved trees shapes the rhizosphere soil fungal communities of coniferous tree species in subtropical forests. For. Ecol. Manag..

[16] Sawada K., Inagaki Y., Sugihara S., Funakawa S., Ritz K., Toyota K. (2021). Impacts of conversion from natural forest to cedar plantation on the structure and diversity of root-associated and soil microbial communities. Appl. Soil. Ecol..

[17] Tripathi B.M., Song W., Slik J.W.F., Sukri R.S., Jaafar S., Dong K., Adams J.M. (2016). Distinctive tropical forest variants have unique soil microbial communities, but not always low microbial diversity. Front. Microbiol..

[18] Kruse S., Wieczorek M., Jeltsch F., Herzschuh U. (2016). Treeline dynamics in Siberia under changing climates as inferred from an individual-based model for *Larix*. Ecol. Model..

[19] Bao T., Deng S., Yu K., Li W., Dong A. (2021). Metagenomic insights into seasonal variations in the soil microbial community and function in a *Larix gmelinii* forest of Mohe, China. J. For. Res..

[20] Song D., Cui Y., Ma D., Li X., Liu L. (2022). Spatial variation of microbial community structure and its driving environmental factors in two forest types in permafrost region of Greater Xing’an Mountains. Sustainability.

[21] Jiang Y., Wu S., Yang L., Liu Y., Gao M., Ni H. (2024). Short-term simulated warming changes the beta diversity of bacteria in taiga forests’ permafrost by altering the composition of dominant bacterial phyla. Forest.

[22] Ade L.J., Hu L., Zi H.B., Wang C.T., Lerdau M., Dong S.K. (2018). Effect of snowpack on the soil bacteria of alpine meadows in the Qinghai-Tibetan Plateau of China. Catena.

[23] Bossio D.A., Scow K.M. (1998). Impacts of carbon and flooding on soil microbial communities: Phospholipid fatty acid profiles and substrate utilization patterns. Microb. Ecol..

[24] Zhang Y., Yao S., Cao X., Schmidt-Rohr K., Olk D.C., Mao J., Zhang B. (2018). Structural evidence for soil organic matter turnover following glucose addition and microbial controls over soil carbon change at different horizons of a Mollisol. Soil Biol. Biochem..

[25] Ma T., Zhu S., Wang Z., Chen D., Dai G., Feng B., Su X., Hu H., Li K., Han W. (2018). Divergent accumulation of microbial necromass and plant lignin components in grassland soils. Nat. Commun..

[26] Albers D., Migge S., Schaefer M., Scheu S. (2004). Decomposition of beech leaves (*Fagus sylvatica*) and spruce needles (*Picea abies*) in pure and mixed stands of beech and spruce. Soil Biol. Biochem..

[27] Gruba P., Mulder J. (2015). Tree species affect cation exchange capacity (CEC) and cation binding properties of organic matter in acid forest soils. Sci. Total Environ..

[28] Ross D.S., Matschonat G., Skyllberg U. (2008). Cation exchange in forest soils: The need for a new perspective. Eur. J. Soil. Sci..

[29] Clarholm M., Skyllberg U. (2013). Translocation of metals by trees and fungi regulates pH, soil organic matter turnover and nitrogen availability in acidic forest soils. Soil Biol. Biochem..

[30] Van Nevel L., Mertens J., De Schrijver A., Baeten L., De Neve S., Tack F.M., Meers E., Verheyen K. (2013). Forest floor leachate fluxes under six different tree species on a metal contaminated site. Sci. Total Environ..

[31] Zhou Q., Keith D.M., Zhou X., Cai M., Cui X., Wei X., Luo Y. (2018). Comparing the water-holding characteristics of broadleaved, coniferous, and mixed forest litter layers in a karst region. Mt. Res. Dev..

[32] Rivero R.G., Grunwald S., Osborne T.Z., Reddy K.R., Newman S. (2007). Characterization of the spatial distribution of soil properties in water conservation area 2A, Everglades, Florida. Soil Sci..

[33] Deng J., Zhu W., Zhou Y., Yin Y. (2019). Soil organic carbon chemical functional groups under different revegetation types are coupled with changes in the microbial community composition and the functional genes. Forests.

[34] Tian S., Man X. (2016). Characteristics of soil microbial biomass carbon and dissolved organic carbon in northern forest region of Daxing’an Montains. Chin. J. Soil Sci..

[35] Broadbent A.A.D., Newbold L.K., Pritchard W.J., Michas A., Goodall T., Cordero I., Giunta A., Snell H.S.K., Pepper V.V.L.H., Grant H.K. (2024). Climate change disrupts the seasonal coupling of plant and soil microbial nutrient cycling in an alpine ecosystem. Glob. Change Biol..

[36] Kafle A., Cope K.R., Raths R., Yakha J.K., Subramanian S., Bücking H., Garcia K. (2019). Harnessing soil microbes to improve plant phosphate efficiency in cropping systems. Agronomy.

[37] Ji Y., Zhang P., Shen H. (2023). Competition intensity affects growing season nutrient dynamics in Korean pine trees and their microhabitat soil in mixed forest. For. Ecol. Manag..

[38] Qi J., Liu Y., Wang Z., Zhao L., Zhang W., Wang Y., Li X. (2021). Variations in microbial functional potential associated with phosphorus and sulfur cycling in biological soil crusts of different ages at the Tengger Desert, China. Appl. Soil Ecol..

[39] Atiwesh G., Parrish C.C., Banoub J., Le T.T. (2022). Lignin degradation by microorganisms: A review. Biotechnol. Prog..

[40] Zhu W., Hao M., Zhao W., Yu S., Fan Z., Liu Y., Dun X., Zhang Z., Gao P. (2025). Changes of microbial life history strategies to soil nutrient limitations following vegetation restoration and its impact on carbon utilization efficiency. J. Environ. Manag..

[41] Guo Y., Liu X., Tsolmon B., Chen J., Wei W., Lei S., Yang J., Bao Y. (2020). The influence of transplanted trees on soil microbial diversity in coal mine subsidence areas in the Loess Plateau of China. Glob. Ecol. Conserv..

[42] Rodriguez D.R.A., Scheu S., Rillig M.C. (2025). Soil microbial responses to multiple global change factors as assessed by metagenomics. Nat. Commun..

[43] Brown R.W., Chadwick D.R., Bending G.D., Collins C.D., Whelton H.L., Daulton E., Covington J.A., Bull I.D., Jones D.L. (2022). Nutrient (C, N and P) enrichment induces significant changes in the soil metabolite profile and microbial carbon partitioning. Soil Biol. Biochem..

[44] Heděnec P., Nilsson L.O., Zheng H., Gundersen P., Schmidt I.K., Rousk J., Vesterdal L. (2020). Mycorrhizal association of common European tree species shapes biomass and metabolic activity of bacterial and fungal communities in soil. Soil Biol. Biochem..

[45] Camenzind T., Hättenschwiler S., Treseder K.K., Lehmann A., Rillig M.C. (2018). Nutrient limitation of soil microbial processes in tropical forests. Ecol. Monogr..

[46] Zhang D., Wang L., Qin S., Kou D., Wang S., Zheng Z., Penuelas J., Yang Y. (2023). Microbial nitrogen and phosphorus co-limitation across permafrost region. Glob. Change Biol..

[47] Liu G., Wang H., Yan G., Wang M., Jiang S., Wang X., Xue J., Xu M., Xing Y., Wang Q. (2023). Soil enzyme activities and microbial nutrient limitation during the secondary succession of boreal forests. Catena.

[48] Guo Y.X., Yu G.H., Hu S., Liang C., Kappler A., Jorgenson M.T., Guo L., Guggenberger G. (2024). Deciphering the intricate control of minerals on deep soil carbon stability and persistence in Alaskan permafrost. Glob. Change Biol..

[49] Theocharis A., Clement C., Barka E.A. (2012). Physiological and molecular changes in plants grown at low temperatures. Planta.

[50] Ernakovich J.G., Wallenstein M.D. (2015). Permafrost microbial community traits and functional diversity indicate low activity at in situ thaw temperatures. Soil Biol. Biochem..

[51] Maillard F., Klinghammer F., Jassey V.E.J., Zhang B., Kennedy P.G., Lara E., Geisen S., Tranvik L., Hammer E., Tunlid A. (2025). Hidden decomposers: Revisiting saprotrophy among soil protists and its potential impact on carbon cycling. Soil Biol. Biochem..

[52] Maillard F., Michaud T.J., See C.R., DeLancey L.C., Blazewicz S.J., Kimbrel J.A., Pett-Ridge J., Kennedy P.G. (2023). Melanization slows the rapid movement of fungal necromass carbon and nitrogen into both bacterial and fungal decomposer communities and soils. Msystems.

[53] Buckeridge K.M., Creamer C., Whitaker J. (2022). Deconstructing the microbial necromass continuum to inform soil carbon sequestration. Funct. Ecol..

[54] Angst G., Angst Š., Frouz J., Jabinski S., Jílková V., Kukla J., Li M., Meador T.B., Angel R. (2024). Stabilized microbial necromass in soil is more strongly coupled with microbial diversity than the bioavailability of plant inputs. Soil Biol. Biochem..

[55] Yang L., Lyu M., Li X., Xiong X., Lin W., Yang Y., Xie J. (2020). Decline in the contribution of microbial residues to soil organic carbon along a subtropical elevation gradient. Sci. Total Environ..

[56] Xie L., Pang X., Yin C. (2025). Soil fungal community composition drives forest-specific and seasonal dynamics of microbial carbon use efficiency in subalpine ecosystems. J. Plant Ecol..

[57] Soong J.L., Fuchslueger L., Maranon-Jimenez S., Torn M.S., Janssens I.A., Penuelas J., Richter A. (2020). Microbial carbon limitation: The need for integrating microorganisms into our understanding of ecosystem carbon cycling. Glob. Change Biol..

[58] Hu J., Du M., Chen J., Tie L., Zhou S., Buckeridge K.M., Cornelissen J., Huang C., Kuzyakov Y. (2023). Microbial necromass under global change and implications for soil organic matter. Glob. Change Biol..

[59] Malik A.A., Puissant J., Buckeridge K.M., Goodall T., Jehmlich N., Chowdhury S., Gweon H.S., Peyton J.M., Mason K.E., van Agtmaal M. (2018). Land use driven change in soil pH affects microbial carbon cycling processes. Nat. Commun..

[60] Song J., Zhang H., Gunina A., Mganga K.Z., Chang F., Yu R., Zhou J., Chen A., Li Y. (2025). Subsurface application of organic ameliorant in saline soils increases microbial necromass accumulation in mineral-associated organic matter. Carbon Res..

[61] Wang B., Dou Y., Liang C., Liu C., Ao D., Yao H., Yang E., An S., Wen Z. (2024). Microbial necromass in soil profiles increases less efficiently than root biomass in long-term fenced grassland: Effects of microbial nitrogen limitation and soil depth. Sci. Total Environ..

